# Audio-based event detection in the operating room

**DOI:** 10.1007/s11548-024-03211-1

**Published:** 2024-06-11

**Authors:** Jonas Fuchtmann, Thomas Riedel, Maximilian Berlet, Alissa Jell, Luca Wegener, Lars Wagner, Simone Graf, Dirk Wilhelm, Daniel Ostler-Mildner

**Affiliations:** 1 Research Group MITI, Klinikum rechts der Isar, TUM School of Medicine and Health, Technical University of Munich, Munich, Germany; 2 Department of Surgery, Klinikum rechts der Isar, TUM School of Medicine and Health, Technical University of Munich, Munich, Germany; 3 University Hospital of Hearing, Speech and Voice Disorders, Medical University of Innsbruck, Innsbruck, Austria

**Keywords:** Audio signal classification, Event detection, Workflow analysis, Surgical sound analysis

## Abstract

**Purpose:**

Even though workflow analysis in the operating room has come a long way, current systems are still limited to research. In the quest for a robust, universal setup, hardly any attention has been given to the dimension of audio despite its numerous advantages, such as low costs, location, and sight independence, or little required processing power.

**Methodology:**

We present an approach for audio-based event detection that solely relies on two microphones capturing the sound in the operating room. Therefore, a new data set was created with over 63 h of audio recorded and annotated at the University Hospital rechts der Isar. Sound files were labeled, preprocessed, augmented, and subsequently converted to log-mel-spectrograms that served as a visual input for an event classification using pretrained convolutional neural networks.

**Results:**

Comparing multiple architectures, we were able to show that even lightweight models, such as MobileNet, can already provide promising results. Data augmentation additionally improved the classification of 11 defined classes, including inter alia different types of coagulation, operating table movements as well as an idle class. With the newly created audio data set, an overall accuracy of 90%, a precision of 91% and a *F*1-score of 91% were achieved, demonstrating the feasibility of an audio-based event recognition in the operating room.

**Conclusion:**

With this first proof of concept, we demonstrated that audio events can serve as a meaningful source of information that goes beyond spoken language and can easily be integrated into future workflow recognition pipelines using computational inexpensive architectures.

## Introduction

Workflow analysis in the operating room has been subject to research for many years [1–3]. Continuously collecting information about the ongoing surgery is essential to create a digital representation of the current procedural tasks, the surgical phase, and overall situation. Almost all kinds of sophisticated assistive technologies that are foreseen to actively participate in surgical procedures in the future rely on the creation of a digital representation of the intervention [1, 3]. If, for example, a cognitive OR should dim the lights on time when a laparoscopic phase starts, or an autonomous-acting robotic circulator should fetch sterile goods when additional material is needed, the reliable situation awareness of a digital system is an essential prerequisite.

In order to create a system of such kind, previously proposed concepts mainly relied on the combination of multiple information sources by integrating signals of various surgical devices for the recognition of patterns, e.g., the activation of energy devices or the movement of an operating table [4, 5]. However, a major challenge is that today’s OR hardware lacks standardized communication protocols and interfaces. Moreover, possible data acquisition mostly relies on actual physical inter-device connections, which is cumbersome in the OR environment, as devices are often shared and moved between several OR suites and additional cables have to be run on the floor. Hence, comprehensive data acquisition setups can mainly be found in research projects and are rather a mere juxtaposition of workarounds, than a holistic solution with access to all devices at the push of a button. Efforts are made by consortia like OR.net to harmonize interfaces of upcoming products, and the first demonstrators of connected operating rooms such as the *IVAP2025* in Munich, Germany, or the *SCOT* in Tokyo, Japan, have been presented [4, 6, 7]. Unfortunately, those setups are individual implementations that go along with high original costs and will ultimately not be integrated into an extensive number of ORs in the foreseeable future. This, however, contrasts with the need for multicentric large-scale data acquisition, which is required for building robust and reliable cognitive OR systems.

Due to the advancements in machine learning techniques in recent years, focus in research on workflow analysis shifted heavily to the processing of intraoperative images as an essential component. Instrument detection within laparoscopic videos or a semantic understanding of the surgical scene from the outer perspective using RGB(-Depth) cameras strongly contributed to the progress of workflow analysis [8, 9]. The main drawbacks of these video-based approaches are the need for a high-quality image as well as the diverse look and privacy concerns. With only a fraction of today’s interventions being performed minimally invasively, the availability of intraabdominal video signals can also not be taken for granted [10]. On the contrary, outer cameras require at least a direct line of sight, which turns out to be challenging in real-life OR environments due to multiple equipment booms and moving staff obstructing the view [11–13]. Especially in larger scenes, smaller objects can be hard to detect, and the high dynamic lighting situation poses an additional difficulty.

While all concepts contributed substantially to this field, an optimal solution for a simple yet robust data acquisition setup has not been found so far. In the following, we therefore propose a new way of collecting intraoperative data using the previously neglected dimension of audio. This proof of concept thereby combines both aforementioned approaches by gathering information from a diversified set of medical devices—via their sounds—in conjunction with well-established deep-learning-based image classification techniques transferred to the domain of audio.

## Methodology

As the literature has proven, gathering status information from a larger set of surgical devices can be sufficient for phase detection, even without the use of any video signals [4, 5]. Building upon this idea, but overcoming the cumbersome process of establishing an individual connection to each and every device, we base our setup merely on audio signals. During an intervention, the visual and haptic perception of surgeons is already utilized to capacity. That is why sound design of technical equipment is an important part of the development process of almost every device nowadays. Audio icons are created to provide characteristic feedback of current settings and deployment status for the OR staff. Capturing those signals via microphones equals the auditory perception of a surgeon and reduces a complex sensor setup to a low-cost, potentially even wireless, solution that is spatially flexible and does not require any line of sight. Apart from medical devices and their signature sound, other distinct environmental sounds, such as the opening of wrapped sterile instruments, the mechanical noise of table movements, or the opening and closing of doors, can be used as well to spot process-related events.

In the following, we demonstrate these possibilities in two steps. First, we describe the creation of a data set and the recording setup in the operating room. Second, we explain the computational processing of the data.

### Data acquisition

Despite extensive research in the field of surgical data science, no publicly available, broad collection of surgical audio recordings exists. That is why, as an initial step, creating a high-quality audio data set was inevitable.

**Fig. 1:**
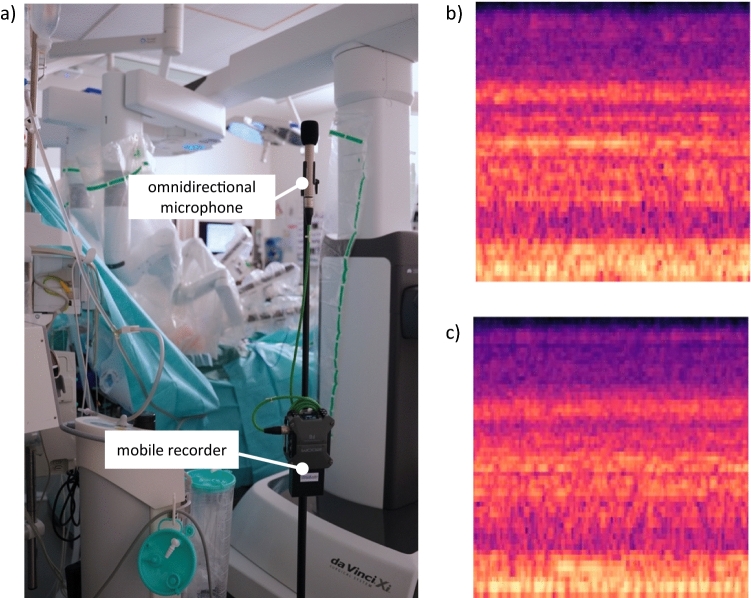
**a** shows the recording setup within the operating room with the microphones in proximity to the patient’s head. **b** and **c** Depict spectrograms in their final form, ready for the analysis using a CNN. While **b** represents an operating table moving down, **c** represents the sound of the operating table moving up

Recording took place in multiple operating rooms of the University hospital rechts der Isar, in Munich, Germany, during 23 surgeries. The interventions comprised laparoscopic hemicolectomies and sigmoid colectomies performed on a robotic system (Da Vinci Xi, Intuitive Surgical Inc., Sunnyvale, USA), thus limiting the set of used devices. The setup consisted of two microphones, placed onto tripods. Proximity to the patient's head was striven for, as this position is quite often close to the center of the room and is subsequently a sweet spot for universal audio capturing. However, the actual position was usually in a one meter distance, due to the surrounding equipment, such as IV lines or sterile drapes. Figure 1a shows the setup within the operating room. While an omnidirectional condenser microphone (NT-55, RØDE Microphones, Silverwater, Australia) was capturing general audio signals from all directions, an additional cardioid condenser microphone (NT-45, RØDE Microphones, Silverwater, Australia) was directed at specific regions of interest such as the surgical site. Solely sounds of the operating table were recorded separately within close distance to the table’s column during a series of interventions. Captured audio signals were then recorded with a sample rate of 44.1 kHz in 32-bit using a portable audio recorder (Zoom F6, Sound Service GmbH, Berlin, Germany). All recorded surgeries were annotated live by medical students, starting from the preparation of the instruments by the nurses until the patient left the OR. Thereby, timestamps of characteristic audio events in relation to the clock of the recording device were manually notated for easier sample retrieval in the postprocessing and identification of non-distinct sounds. Additionally, notes regarding the current surgical phase and the overall situation in the OR were made, describing, for example, whether the radio was running or if additional guests were talking in the background.

After the successful capture, whole recordings were cut into small snippets of one-second length. The snippets were normalized regarding their volume and manually labeled. The short length allowed the assignment of a single label for each file. Thereby, 11 classes of sound events were defined as listed in Table 1, with an idle class comprising background sounds. For this proof of concept, we limited the scope of audio recognition to five devices while in return, differentiating between multiple intra-device settings. Here, the objective was to demonstrate the functionality for a wide variety of sounds—from ringing phones in the background to specific instrument subtypes—while also testing rather challenging differentiations, like individual surgical table movements. The chosen categories thereby also align with requirements for a first coarse phase detection, as several sounds mark distinctive events within the OR. For example, when the patient is transferred into the OR, the column will be moved all the way up to mount and lock the surgical table top from the shuttle. When the first use of monopolar coagulation is registered, it indicates that the surgery has started, as the monopolar coagulation is initially used for hemostasis after the cut into the well-vascularized skin.

**Table 1:** Overview of the defined classes and their labels, as well as the number of collected samples per class and the amount used for training including augmented samples

Class Label	Collected Samples	Original samples used for the second step training	Augmented samples used for the second step training
Monopolar Coagulation	8377	700	–
Bipolar Coagulation	1064	700	–
Monopolar Cutting	253	–	–
Ultrasound Dissector	778	477	223
Table up	1282	700	–
Table down	1284	700	–
Table tilts	1171	700	–
Table back/forth	991	691	9
Phone ringing	516	216	484
DaVinci Sounds	934	634	66
Idle	5037	700	–

### Data processing

For future live detection of audio-based events during an intervention, gathered audio samples were preprocessed and subsequently used to train a deep learning-based classification pipeline.

The sample size of the different classes was quite unbalanced due to some events such as coagulation occurring way more often than others. Hence, in a first step samples of smaller classes were augmented on the raw audio level to increase their sample size using, e.g., various levels of added noise.

In order to make the audio signals accessible for the classification task itself, we converted the waveforms into spectrograms, as spectrograms are today’s dominant feature for audio processing in deep learning [14]. They are a two-dimensional visual representation of an audio sequence and depict the temporal course of the signal on the abscissa, individual frequencies on the ordinate, and the signal amplitude as a color code with brighter colors representing louder frequencies. By applying a rectangular sliding window function to the individual samples in the data set, we computed log-mel-spectrograms based on a short-time Fourier transformation and a filter bank with 64 filters.

As humans perceive frequencies not on a linear scale, the mel-scale creates a representation where frequencies that are equidistant for a listener are equidistant in a spectrogram [15, 16].

The window length was thereby set to 2048 data points with a continuous overlap of 75%, resulting in 46.44 ms long samples as presented in Fig. 1b and c.

Since the higher frequencies contained mostly noise, which decreased the overall classification accuracy in initial test runs, frequencies above 12 000 Hz were cut off and the spectrograms were readjusted in their range of amplitude.

Preprocessed spectrograms were subsequently used as an input for transfer learning with classical convolutional neural networks. Networks were implemented using Tensorflow [17], Keras [18] and pretrained weights based on ImageNet [19]. Results presented in the section below were achieved using a 70%/15%/15% split for all training/validation/test purposes.

## Results

In a two-step approach, first, a series of network architectures were evaluated, using a smaller subset of 200 samples per class. Results of this first step are provided in Table 2 and were achieved with a batch size of 32. While some networks learned hardly any features, others like the *Inception V3* [20], with an overall test accuracy of 81%, or the *MobileNet* [21], with an overall accuracy of 88%, performed well. Even though *DenseNet169* [22] and *MobileNet* provided comparable results, they differed in their prediction speed with *MobileNet* being more than three times faster than *DenseNet169* and thus being more suited for real-time applications.

**Table 2:** Results for multiple architectures of pretrained networks, using a small subset of 200 training samples for 11 classes

Architecture	Accuracy	Precision	Recall	*F*1-score
EfficientNetB4 [24]	0.09	0.0083	0.009	0.02
InceptionV3 [20]	0.81	0.81	0.81	0.81
ResNet50 [25]	0.51	0.50	0.51	0.50
VGG-19 [26]	0.78	0.79	0.79	0.79
MobileNet [21]	0.88	0.89	0.88	0.89
DenseNet169 [22]	0.88	0.88	0.88	0.88

**Fig. 2:**
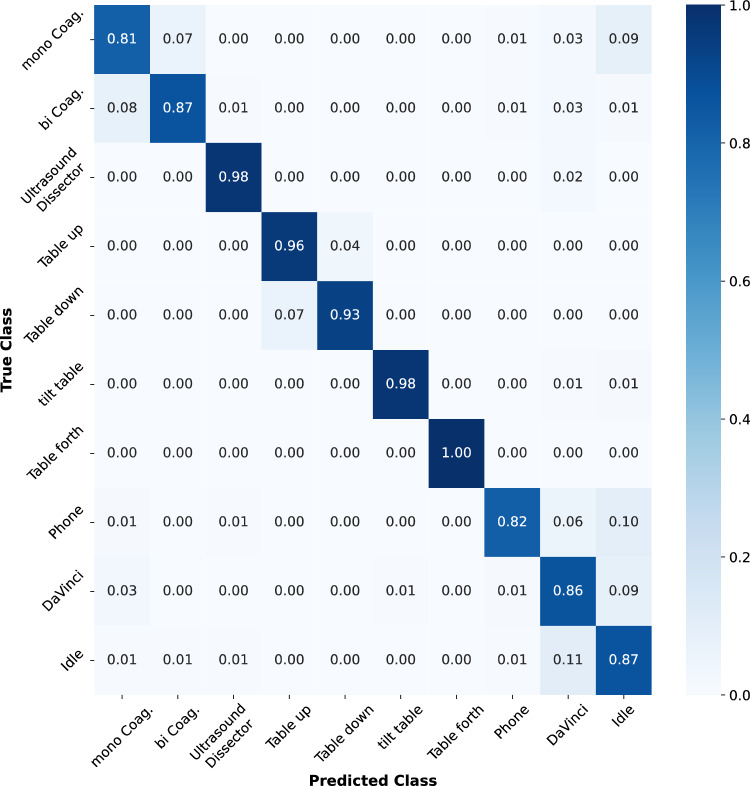
Balanced and normalized confusion matrix for the MobileNet architecture, trained with 700 samples for each of the 10 classes. Due to rounding errors, the sum of certain rows exceeds 1.0

Hence in the second step, we chose *MobileNet* for an in-depth optimization using the full data set of 700 training samples per class. Despite more than 63 h of recorded audio, the quantity of acquired samples was not sufficient for the purpose of training with all defined classes. Therefore, the class *monopolar cutting* was excluded for this second step, as the amount of available data, with 253 samples, was significantly lower than other classes. Whenever the amount of available training samples was close but lower than the target of 700, additional samples were created via augmentation techniques as mentioned in the methodology section. Hyperparameter optimization ultimately resulted in an average accuracy for the test set of 90 ± 0.4%, an average precision of 91 ± 0.25%, a recall of 91 ± 0.35%, and an *F*1-score of 91 ± 0.29%, with the standard deviations based on 10 individual trained networks. Figure 2 shows the normalized and balanced confusion matrix for the 10 defined classes. Additional improvements up to an accuracy of 92 ± 0.26% were observed when dropping the class *phone*, which was based to a large extent on augmented samples.

In the predictions, the network seems to slightly mix up the two coagulation classes with each other, as well as the table movements up and down. The audio comments of the DaVinci Robot that form a quite diverse group of samples within its own class get sometimes confused with the idle class that also contains spoken language of the medical staff.

## Discussion

As a proof of concept, this initial proposal is meant to introduce the domain of audio as a valuable source of information for future workflow analysis systems in the OR. Even though the pipeline was kept quite general and simple on purpose, results are very promising. 

Within the scope of this paper, OR conditions were taken as given for the recordings. Thus, there is a lot of background noise on the data set, ranging from air-conditioning to radios playing in the background. Here, digital filtering in the preprocessing step could further improve future results. In an advanced setup, the positioning of microphones can also be optimized, thereby physically reducing the dominance of individual audio sources that have been close to the current microphone’s position, such as the suction device on Fig. 1a. Hence, microphones mounted at the OR’s ceiling, even as an array for added spatial resolution, should be evaluated.

For this initial proof of concept, we limited the scope to 10 different events that can help to identify the timestamps currently manually documented by the circulator, such as a patient entering the OR or the start of a surgery. However, for an actual deduction of surgical phases, the data set has to be extended to a more comprehensive list of entities. In conjunction with a phase annotation, patterns in the temporal relation of individual audio events can then be analyzed and surgical phases identified via a second classification step.

Also, an optimal sample length as a tradeoff between real-time capability and precision is yet to be evaluated. Furthermore, the presented event classification only relies on classic CNNs in order to provide some baseline results and prove the feasibility of event recognition using inexpensive computational methods for a later implementation on edge devices. However, more advanced machine learning techniques, such as transformer networks, could further improve the results and include temporal information.

From a medical point of view, our setup has the advantage of not being limited to a defined set of medical devices equipped with sensors or dedicated software interfaces but being deployable in every possible OR configuration and device combination. Even though direct data from medical devices would be preferable in the long run, audio recognition can contribute to the fast scaling of workflow data libraries in the research community already today. It even goes beyond the devices and can recognize distinct environmental sounds like dropping instruments for example or the unwrapping of sterile draping. This advantage is even amplified by the fact that a created data set is not limited to the training of one type of intervention only, such as widespread image-based datasets like *Colec80* [23] for cholecystectomies. As the audio data itself is universal, it can be reused as training data in all kinds of interventions and even across various surgical disciplines. Furthermore, the sound signatures are completely patient-independent and do not relate to age, sex, or ethnicity. As all predictions can run offline on edge devices, due to the lightweight audio data, confidential information does not need to leave the OR and can be processed as a stream with only identified events being logged.

While the temporal resolution of phase detection, entirely based on audio events, has yet to be evaluated, we are convinced that the combination with other modalities and techniques such as key word recognition would elevate current workflow detection pipelines to the next level in terms of prediction accuracy.

## Conclusion

With an intelligent OR in mind, we presented a new approach for the future development of workflow recognition systems by incorporating audio signals. Microphones as a low-cost sensor in conjunction with log-mel-spectrogram-based signal analysis using deep convolutional neuronal networks showed promising results, when applied on a unique data set of more than 20.000 individually gathered OR audio samples. Using the *MobileNet* network, we were able to achieve up to 90% accuracy for the recognition of 10 classes, including audio events such as directed table movements or instrumentation with specific energy devices.

Next, we plan to expand our data set, including the variety of detectable events, and connect single predictions with a temporal context model. Meanwhile, we encourage the community to build upon this new approach and consider the dimension of audio as a highly informative data source for future workflow recognition systems.
